# “We Tried to Borrow Money, but No One Helped.” Assessing the Three-Delay Model Factors Affecting the Healthcare Service Delivery among Dengue Patients during COVID-19 Surge in a Public Tertiary Hospital: A Convergent Parallel Mixed Methods Study

**DOI:** 10.3390/ijerph182211851

**Published:** 2021-11-12

**Authors:** Antonio D. Ligsay, Maurice Lee B. Santos, Epifania S. Simbul, Kristan Jela M. Tambio, Michelle Joyce M. Aytona, Grecebio Jonathan D. Alejandro, Richard Edward L. Paul, Zypher Jude G. Regencia, Emmanuel S. Baja

**Affiliations:** 1 The Graduate School & College of Science, University of Santo Tomas, Manila 1008, Philippines; gdalejandro@ust.edu.ph; 2 Clinical Research Section, St. Luke’s College of Medicine-William H. Quasha Memorial, Quezon City 1112, Philippines; 3 National Children’s Hospital, New Manila, Quezon City 1113, Philippines; santosmauricelee@gmail.com (M.L.B.S.); esimbul@yahoo.com (E.S.S.); kristan.tambio@gmail.com (K.J.M.T.); mjaytona@gmail.com (M.J.M.A.); 4 College of Nursing, Manila Tytana Colleges, Metropolitan Park, Pasay City 1300, Philippines; 5 Functional Genetics of Infectious Diseases Unit, Institut Pasteur, UMR 2000 (CNRS), 75015 Paris, France; richard.paul@pasteur.fr; 6 Institute of Clinical Epidemiology, National Institutes of Health, University of the Philippines Manila, Ermita, Manila 1000, Philippines; zgregencia@up.edu.ph (Z.J.G.R.); esbaja@up.edu.ph (E.S.B.); 7 Department of Clinical Epidemiology, College of Medicine, University of the Philippines Manila, Ermita, Manila 1000, Philippines

**Keywords:** delay to care, dengue, healthcare service delivery, Philippines, three-delay model

## Abstract

Identification of delay barriers to care is essential for an effective and efficient healthcare service delivery. In this study, we described the delay in care among parents of the patients seeking treatment for dengue. We also examined the factors affecting the severity of dengue (dengue with warning signs; severe dengue). A convergent parallel design mixed-method approach using Key Informant Interviews (KII) and a survey guided by the Three-Delay Model were conducted among 24 respondents at the National Children’s Hospital (NCH). Coding and thematic analysis using NVIVO and bivariable generalized linear models with a Poisson distribution and robust variance were utilized to analyze the KII transcripts and survey data, respectively. Results showed that financial constraints and previous dengue infection (first delay), mode of transportation, traffic density, and location (second delay), and hospital capacity (third delay) influenced the overall delay uncertainty in seeking care treatment for dengue infection. Furthermore, our bivariable analysis showed that travel time to NCH and place of residency, service given from previous health facilities, and parents’ educational background were associated and played a role in the severity of dengue infection. Interventions focused on the identified factors contributing to delayed care should be made to avoid unwanted clinical outcomes.

## 1. Introduction

Dengue is a mosquito-borne viral disease that has drastically increased in incidence worldwide over the last few decades, currently infecting an estimated 390 million people annually and resulting in an approximated 20,000 deaths [1]. In addition to the burden of morbidity and mortality, it is a significant public health problem because of its economic impact on the health system and families [2]. People living in the low- and lower-middle-income countries such as the Philippines acutely experience these adverse effects of the burden of dengue disease; despite the continued efforts to control the mosquito vectors, there are still dengue outbreaks [3].

According to the Epidemiology Bureau of the Department of Health of the Philippines, 271,480 dengue cases were registered from January to August 2019, which is 95% greater compared to the count of 85,981 of the same period in the previous year [4]. In addition, it is reported that Quezon City, Philippines, had over 3000 cases of dengue, which is the highest in the National Capital Region (NCR) [5]. Defogging and cleanup drives are usually implemented to reduce the incidence of dengue in areas reporting a high incidence of the disease. However, for these interventions to be effective, these urban areas need to analyze and evaluate their current strategies on dengue control and strengthen their dengue control measures.

In addition to vector control strategies and information campaigns to educate the population on best practices for environmental hygiene and elimination of breeding sites, it is also necessary to identify any underlying problems in the health service delivery functions. These issues are especially pertinent to sprawling megacities in the tropics, where unhygienic urbanization enables the proliferation of dengue [6,7,8]. There are four recognized barriers to the utilization of healthcare services: service system (availability, cost, waiting system, and urgency of appointment), structural or physical barriers (cleanliness, the distance of medical facility, and transportation), equipment and medication (availability and cleanliness), and staff competency (lack of knowledge, medical errors, ratio, personal characteristics, and connection) [9].

The three-delay model, commonly used in Obstetric and Gynecological studies, is a model that best describes the delay or barriers to health [10,11]. The first delay involves the onset of a complication and acknowledging the need to transport the patient to a health facility. This delay is a combination of several factors that can lead the patient not to seek prompt treatment from a health facility with the capacity to manage complications [12]. Further delay may have been caused by the families’ perceived poor quality of care. In addition, reported experiences of health personnel’s mistreatment have been documented.

Moreover, limited privacy and a lack of personal care at the delivery facility are likely to occur [13]. The second delay is the elapsed time between departing from home and reaching the facility. It can be, for example, due to transportation issues or financial constraints. Additionally, the third delay involves the elapsed time from the presentation at the facility to the requirement of appropriate treatment [14,15] due to both patient and health worker factors [16]. These identified types of delay contribute to barriers to access to healthcare. Therefore, it is essential to describe these barriers to determine the existing health service access and delivery gaps. It will be helpful particularly for informing the government regarding healthcare services, as well as proper identification of opportunities for future research [17]. These significant barriers are well described in the literature, including, but not limited to, weather and seasonal conditions [18,19,20], limited available transportation [21,22,23], cost of travel [24,25,26], and healthcare provider-related barriers [27,28,29].

While well studied in Obstetrics and Gynecology, no researchers, to our knowledge, utilize the Three-Delay Model regarding tropical diseases. Therefore, in this study, we aim to describe the delay to care among parents of patients seeking treatment for dengue infection in Quezon City, the Philippines. Furthermore, using the Three-Delay Model, we also aim to determine and estimate the effect of the time-delay factors on the severity of dengue infection of the patients.

## 2. Materials and Methods

### 2.1. Study Design and Setting

Since this is the first study that looked at the delay in decision-making among parents of patients with dengue infection, a mixed methods study utilizing convergent parallel design was used to capture and ensure that the findings are grounded in the parents of the patients’ experiences. In addition, we used semi-structured Key Informant Interviews (KII) for the qualitative data collection component of the study and a structured questionnaire survey for the quantitative data collection.

The study was carried out at the National Children’s Hospital (NCH), located at Quezon City, a special tertiary and training hospital under the Department of Health (DOH) in the Philippines that provides care to children. Data collection was ascertained from February to March 2021 during the COVID-19 community quarantine restrictions in Metro Manila. The study received ethical clearance from an independent research ethics committee and followed the Philippine Data Privacy Act 2012.

### 2.2. Sampling Population and Sample Size

The study involved parents of the patients admitted at NCH with a clinical diagnosis of dengue infection. All parents of the patients aged 0 to 17 years old admitted to the hospital during February and March 2021 and can understand the English or Filipino languages were asked to be included in the study. A total of 24 parents, who fulfilled the inclusion criteria, were included in the study. An informed consent process was carried out to allow the participants to be given sufficient time to read and understand the informed consent. After obtaining informed consent, the research assistants interviewed the participants for the KII and administered the quantitative survey.

### 2.3. Data Collection

KII was administered in English or Filipino to obtain knowledge and discern relevant themes regarding the factors that affect the parents’ decision-making process in health-seeking behaviors, particularly the delay to care. Topic guide questions focused on the three different delay segments of the modified Three-Delay Model. Moreover, these questions ask for parents’ thoughts about their child’s sickness before the patients arrived at NCH; home care practices such as traditional medications and religious customs, and medicines bought without prescriptions during illness. In addition, the sequence and timing of homecare practices, the duration of symptoms before seeking care, the signs observed as the main reason for seeking care, and the name and location of the previous healthcare facility to which the patient visited if applicable were asked for. All interviews from the participants were recorded and transcribed.

A questionnaire-based survey followed after the KIIs of the parents. The survey questionnaire was adapted from the Three-Delay Model for Obstetrics [30] to ascertain factors related to health-seeking behaviors due to dengue virus infection. The questions included socio-demographic and clinical information, including signs and symptoms, to classify the severity of the disease. The socio-demographic variables covered age, sex, residence, highest educational attainment, occupation, housing situation, religion, and average household income.

#### 2.3.1. Exposure Assessment

There are no specific guidelines set for the Three-Delay Model regarding the particular amount of time that qualifies as a first, second, or third delay in the literature. However, even a brief amount of time could be classified as a delay since it increases the time between symptom onset and disease management [31]. Here, the participants were asked to state the amount of time (in hours) that was spent between the beginning of the first symptoms of dengue and when they left to seek medical help (first delay), the amount of time spent to reach the hospital (second delay), and the amount of time spent waiting to get the proper treatment from the hospital staff (third delay). Data were recorded as a continuous variable. Time in the delay to seek care was classified into different categories: decision time delay in proceeding to the health facility (<1 h; ≥1 h), service delivery delay at the previous health facility (immediately/within 30 min; >30 min; >60 min; no service given), travel time delay to the current health facility (<1 h; 1 h to <2 h; ≥2 h), and general travel time to the current medical facility (<30 min, 30 min to <60 min; 60 min to <120 min; ≥120 min).

#### 2.3.2. Outcome Measurements

Patients were grouped according to their severity of dengue virus infection (dengue with warning signs; severe dengue) according to the presence of signs and symptoms as defined by the World Health Organization (WHO) [32]. Patients with dengue and warning signs have abdominal pain, vomiting, rapid decrease in platelet count, and require strict observation and medical intervention. Patients with severe dengue have potentially fatal complications due to plasma leaking, fluid accumulation, respiratory distress, severe bleeding, unstable vital signs, and admission to the Intensive Critical Unit (ICU).

### 2.4. Data Analysis

#### 2.4.1. Qualitative Data

Transcripts from the KII were analyzed using NVivo 1.4.1 software [33]. The researchers reviewed the KII transcripts several times to identify mistakes and transcription errors and obtain a general sense of the data. Coding to broad nodes (themes) based on the KII guide questions was done. Identifying differences between specific information to identify both common and divergent themes then followed. Furthermore, we compared and contrasted the different nodes cross-referenced with the KII respondent [34,35,36]. Participants’ selected quotes were included to illustrate the main themes, and pseudonyms were used to present these quotes.

#### 2.4.2. Quantitative Data

Descriptive statistics for the patients’ socio-demographic characteristics were calculated. In addition, associations between every ascertained covariate and the severity of the dengue infection (dengue with warning signs; severe dengue infection) were estimated using separate bivariable generalized linear models (GLMs) with a Poisson distribution, log link function, and a robust variance, which is an appropriate method for cross-sectional studies with common outcomes [37,38,39,40].

Crude (CPR) prevalence ratio with a 95% confidence interval (95% CI) was used to report the effect size estimates for the effect of the various time delays and other factors on the severity of dengue infection of the patients. STATA 17 software (www.stata.com/stata17/; accessed on 20 September 2021) was used to carry out all statistical analyses.

## 3. Results

Twenty-four (24) dengue patients were included in the study. Characteristics of both the patients and the parents are presented in Table 1. The parents had an average age (±SD) of 33.8 (±8.3) years old, did not go to college (63%), and were primarily residents of Quezon City (63%). The majority of the patients belonged to the 0 to 12 years old age bracket (67%) and were females (63%). In terms of socioeconomic status, most of the patients belonged to a low socioeconomic class (68%), as depicted by low average household income (PhP 0–10,000.00).

### 3.1. Three-Delay Model Themes

**Figure 1:**
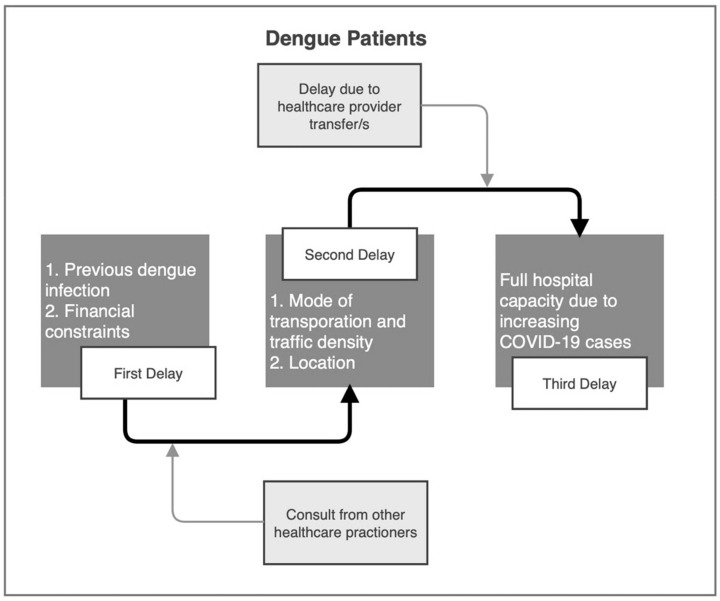
Three-Delay model for the qualitative assessment of delay to care among parents of the patients seeking treatment for dengue infection.

All 24 KII transcripts were analyzed guided by the Three-Delay Model to generate prevailing themes. Figure 1 presents the generated factors that influenced the delay to care among parents of the patients seeking treatment for dengue infection.

#### 3.1.1. First Delay

Most parents of patients experienced the first delay due to the inability to cover the costs that any hospitalization or medical treatment may incur. This factor led to their hesitancy to bring their child to the hospital for treatment. Nevertheless, once the patients’ symptoms began to look worse, most parents immediately brought their child to the nearest health facility. On the other hand, previous dengue infection of patients hastened the decision-making of some of the parents during their child’s illness.

“Before we go to the hospital, we did have to contact somebody from the family and ask for help for our finances before we got admitted.”—SD002

“I was not present when the symptoms first appeared. My aunt called me on the phone and asked me to care for my cousin while she was out. When I got there, I had this suspicion that he might have gotten dengue because it was the same with me when I had it. (…) It was around noon when we decided to bring him to the clinic.”—SD009

“Since this was her second time to have dengue, I am already familiar with the signs and symptoms. When her fever started, I started monitoring her condition and gave her paracetamol every two hours. (...) When her gums started bleeding that same day, my husband and I decided to bring her to the nearest hospital.”—SD013

“I already thought that it was a case of dengue. This situation was my daughter’s second time getting it, so I immediately brought my daughter to see a doctor. (...) I guess finding a way to have the money for hospitalization caused some delay. I tried to find someone who could lend me some money, but no one did. I asked my neighbor for advice, and she told me to go here (NCH).”—SD008

Some parents also opted to bring their child to traditional and alternative, and other healthcare providers such as barangay health workers.

“Evening of that day, she started to have a fever until the next day. We called the “albularyo (folk healer)” to have her checked, and he said, “Naipit Yung ugat (A vein was stuck)” He applied herbal medicine and “hilot (massage).” She did not improve, and she still had a high fever.”—SD012

“I already thought it may be dengue because it was similar to what his sibling experienced, minus the bleeding. I immediately brought him to the Barangay Health Center to get tested for NS1 (dengue antigen). When it turned out to be positive, I brought him here (NCH)”—SD020

#### 3.1.2. Second Delay

This delay was greatly influenced by the availability of transportation, ease of traffic around Quezon City and other parts of Metro Manila, and residency location. Some of the patients lived far from NCH, but some families could immediately bring their children to NCH since they owned private vehicles and were near NCH.

“We rode a tricycle, then a bus, then finally a taxi. It took us one hour to get here. There were many commutes”—SD009

“We left there (barangay health center) at three in the afternoon because we still waited for the results of my daughter’s CBC and Urinalysis and arrived here at five, so approximately two hours. (…) There was a bit of traffic when coming here.”—SD001

“We did not travel that long; in around less than 30 min, we arrived here at NCH. We had a car anyway, so we drove here.”—SD014

“We left Kawit, Kalayaan at three in the afternoon and arrived at San Lazaro Hospital at four. Then went to Jose Reyes Medical Center after San Lazaro. It was near, so it took us less than thirty minutes. Then we went and arrived here at around seven in the evening. We only encountered traffic congestion here in Quezon City (from the City of Manila).”—SD003

“Approximately one hour when we reached NCH. (…). NCH is near our house, so it generally took us about thirty minutes or less to arrive here. We took a tricycle and jeep to come here.”—SD011

#### 3.1.3. Third Delay

The process of leaving their houses and reaching NCH was not well-ordered to some parents and patients. NCH was not their first choice to get proper treatment because it is not located within their area of residence. Most parents opted for NCH because of many hospital refusals due to increasing COVID-19 cases, which led to some hospitals operating at and above total capacity. The parents were also aware that NCH might not immediately administer proper treatment to their child because of COVID-19 cases but, they still waited for their child to get treated even though it took them many hours.

“It is P. Gonzalez Hospital that we went first. It was near us, so that is where we first went. Nevertheless, they told us to go to another hospital because my child might need a blood transfusion, and they did not have a blood bank in the hospital. They referred us to Quirino Memorial hospital, but they could not admit my child when we got there. They said that it is already at full capacity and there is no room left. That is when my husband thought of coming here (NCH). We arrived at night, I think around eight in the evening. It was on Saturday, March thirteen. She was taken care of immediately and placed in the ICU.”—SD017

“We were waiting for 4 h before we got admitted to the ER! My son was already complaining and tired; he was already asking for us just to go home.”—SD004

“I thought they would not be able to take care of her immediately, so we went to Malvar Hospital. When we arrived there, they told us that they do not cater to dengue patients because they lack the facilities, so I searched for another hospital. We went to Diliman Doctors (Hospital) next, but their services were too pricey, so I decided that if we could not find another hospital, I would admit her there and just think about where to get the money to pay them. We took a taxi to get to PCMC (Philippine Children’s Medical Center), but the driver missed the entrance when we got there. We had to circle back, so I asked the driver if there were any other hospitals near us, and he recommended NCH (National Children’s Hospital), so he took us here. We arrived at around two to two-thirty in the afternoon. She was assessed immediately, and the nurses said it was an emergency. They brought her up and was admitted here after the interview (in the ER).”—SD019

“However, on March 19, he still was not improving, so we brought him back to the District Hospital. They tested again for his platelets and CBC, and they said it was low, so they lined him with an IV. Unfortunately, the hospital was full because of COVID-19, so they requested our transfer to another hospital the next day. (…) Unfortunately, we were left to find our hospital. We left at 5 am because they could not accommodate us, so we went to Antipolo Annex, Amang Rodriguez, Rizal Medical, Labor Hospital, but they all cannot accommodate us.”—SD022.

### 3.2. Effect of Delay to Care on Dengue Severity

Table 2 presents the effect of the type of delay and various socio-demographic characteristics of the parents and patients on the severity of dengue infection. Our crude Poisson model shows that patients who traveled to NCH for approximately 1 to 2 h were more likely to have a severe dengue infection by 83% (CPR: 1.83; 95% CI: 1.06–3.18; *p*-value < 0.05) compared to those who only traveled for less than 30 min. As seen in our qualitative data, patients were transferred from one hospital to another due to refusal. The effect of the delay in the previous health facility before NCH was 2.67 times more likely to impact patients and lead to severe dengue infection if they were not given any service (CPR: 2.67; 95% CI: 1.40–5.09; *p*-value < 0.05), compared to patients who were given immediate assistance or within 30 min after arrival. In addition, parents and patients who were non-residents of Quezon City were 2.33 times more likely to get severe dengue infection (CPR: 2.33; 95% CI: 1.03–5.26; *p*-value < 0.05) than those who were residents of Quezon City. Moreover, parents who did not go to college were three times more likely to have a child with severe dengue infection (CPR: 3.00; 95% CI: 0.82–11.02; *p*-value < 0.10) than those parents who went to college.

## 4. Discussion

The drivers for the utilization of the healthcare system depend on socio-demographic factors, educational levels, social structures, gender discrimination, beliefs and practices in certain cultures, economic and political systems, environmental conditions, and natural history of the disease [41,42,43,44,45]. These different factors contribute to the delay or ease of access to care. For dengue infection, it is essential to conduct early diagnosis and give adequate care to manage the disease in order to avoid its development in severe cases [46]. More than two to three days after the onset of dengue symptoms is considered a diagnostic delay associated with the development of shock syndrome and hemorrhagic fever [47]. Similarly, late admission to hospitals is regarded as a predictor of mortality among dengue patients [48]. However, these mentioned studies only dealt with identifying delays at one point in time while seeking care. Through the use of the Three-Delay Model, our mixed-methods study described the delay to care among parents of patients seeking treatment for dengue infection and determined the factors affecting the severity of dengue infection relative to the amount of time of delay.

Our qualitative data collected from the parents of the patients and the quantitative data derived from the survey questionnaire showed different aspects of delaying factors. Both the drivers and barriers of delay to care were assessed through qualitative analysis. Our results showed that the first delay was characterized by previous dengue infection and financial constraints. The former was a barrier to delay, prompting the parents to bring their child to the nearest health facility. History of previous dengue infection has been identified as a factor that can decrease mortality risk which encourages individuals to seek early diagnosis and proper disease management, thus preventing treatment [49].

On the other hand, some parents posited financial constraints as a delaying factor leading to hesitancy in bringing their child to the hospital. A study in another Southeast Asian country reported that most families could not meet the financial resources required for appropriate medical care for malarial infection [50]. It was also observed to be the case in other countries for other diseases [51,52,53,54,55]. More often than not, in those studies, most families tended to self-diagnose and opt for home treatment or traditional and alternative healthcare [50]. Our study reflected the same findings, wherein some parents chose alternative healthcare to treat their children. Most marginalized Filipinos trust the healing abilities of “albularyo/hilot” to alleviate physical and psychological pain [56] as part of cultural practices and beliefs [57]. These “albularyo/hilot” are folk healers, greatly influenced by faith [56]. The limitation of resources can lead to a choice of these treatments, and hence there is a delay in proper treatment from healthcare facilities [58]. This practice can also be attributed to the parent’s level of education. Our quantitative data showed that parents who did not go to college were more likely to have a child with severe dengue infection. The level of education among the parents is vital in creating a difference in preventing delayed or improper healthcare that may lead to unwanted health outcomes [59,60,61]. Our findings provide evidence that parents’ educational level, which also predicts their capacity to pay, plays an essential role in their decision-making in seeking treatment.

The second delay is characterized by available transportation and ease of traffic. The factor identified from the first delay (financial constraint) contributes to the second delay (available transportation). The barrier in transportation for patients brings a considerable disease burden, which reflects the relationship between poverty and available transportation [62]. Estimating the direct impact of transportation barriers on treatment access is difficult, but studies have shown that difficulty in transportation affects healthcare access by 3–6% [63,64]. Similarly, studies also reported that transportation costs negatively affect the decision-making in seeking and adhering to care [65,66,67]. Research also showed no difference between urban and rural transportation barriers, which contributed to care delay [68]. Location of residency and distance from the healthcare facility are also factors of transportation disadvantage. Our quantitative data showed that dengue patients who were non-residents of Quezon City and patients who traveled for 1 to 2 h were more likely to develop a severe infection. Long distances leading to long travel hours contribute to a lack of immediate access to medical facilities since available hospitals are located relatively far from the people who need them, leading to delay in care [69]. Previous research has evaluated the effect of distance as a barrier to care leading to delay, which yielded similar results to those of our study [70,71,72,73,74].

A high refusal rate from the previously visited hospitals was observed from the narratives collected through KII. Most patients suffered from several hospital transfers before reaching NCH as their final treatment facility, which caused a delay in getting and accessing proper medical treatment for the patients. This poor access is attributable to the existing COVID-19 pandemic, which drastically affected hospitals and other healthcare facilities managing COVID-19 patients [75,76]. Hospital spaces were widely dedicated to COVID-19 patients, reducing hospital admission for other patients [77,78,79], including dengue patients, further increasing the third type of delay time. With the pandemic, several studies have suggested that lower hospital capacity due to the COVID-19 surge is related to higher mortality [80]. In our quantitative analysis, as evident from our Poisson model, patients who received no service from previously sought hospitals due to the COVID-19 surge were more likely to develop severe dengue infection than those who received immediate care. Furthermore, improvement in the initial clinical management of patients is always associated with improvement of health outcomes [81].

The reduction of undesirable outcomes, including an increase in severity of the disease or death related to receiving proper care (third delay), needs the control of adequate, timely, and appropriate healthcare facilities [15,82,83], albeit this third delay is directly related to the characteristics of a healthcare facility, including quality of care. In addition, other factors contribute to this delay in receiving appropriate care, such as individual or household barriers [10,84], as seen from the first delay of both qualitative and quantitative data in our study.

### Limitations

Our study employed the convenience sampling method among a small number of respondents, and the study site’s purposive nature limited the study’s generalizability. Further studies using a larger sample size are needed to validate our findings. Different hospitals may also suggest other social health factors in terms of delay to care among their patients. There is a need to acknowledge these social health factors that mutually influence delay to care. Self-reporting of data from the participants, which may contribute to recall bias, should not be ruled out as a source of error. To minimize this source of bias, we carefully tried to question them about the timing of events that led to their hospitalization. In addition, we also guided the participants through a verbal review of the events that had occurred to help them identify and correctly establish the timing of each stage of the process of care-seeking.

The results of our study only apply to dengue patients and their relation to their severity. Future studies may be needed to make further conclusions on the factors affecting delay to care for other diseases. Similarly, data collection happened during the height of the COVID-19 pandemic, which substantially impacted hospitalization capacity. Therefore, the results of our study may be different during a non-pandemic scenario, and further studies are needed to generate evidence for this.

The use of cross-sectional study design in our quantitative component also posits a limitation. Contrasts were mainly between participants from a single-time point; hence, the temporal association between the exposure and the outcome cannot be estimated. In addition, unmeasured or residual confounding factor bias should also not be neglected.

## 5. Conclusions

Our study, being the first in the Philippines that looked at the factors affecting delay to care among dengue patients, has important public health implications. Using the triangulated data of our mixed methods study, we identified the factors that contributed to the delay to care among dengue patients that led to severe dengue complications during COVID-19. By recognizing the barriers that contribute to delay in caring, not only to dengue patients, the government may systematically address these factors. Strengthening existing health education programs and promotions and community mobilization efforts to bring care to the community and prevent delay may be one of the solutions needed to avoid unwanted clinical outcomes. We believe that, to minimize delay to care, the general public should be educated on the symptoms of dengue infection and the importance of early medical consultation. Moreover, through the results of our study, the government may be guided to create an enabling environment with well-functioning health care systems that can give effective and efficient care and treatment in well-equipped facilities managed by skilled healthcare providers.

## Figures and Tables

**Table 1:** Study characteristics (n = 24).

Characteristics ^a^	Total (n = 24)
Age of parents, years (mean (std. dev.))	33.8 (8.3)
Age of patient	
0–12 years old	16 (66.7%)
13–18 years old	8 (33.3%)
Sex of patient	
Female	15 (62.5%)
Male	9 (37.5%)
Non-Quezon city residence	
Yes	9 (37.5%)
No	15 (62.5%)
Relationship status of parent	
Single	12 (50.0%)
In a relationship	12 (50.0%)
Education of parent	
None to high school graduate	15 (62.5%)
At least a college student	9 (37.5%)
Religion	
Catholic	12 (50.0%)
Non-Catholic	12 (50.0%)
Occupation of parent	
None/Housewife	11 (45.8%)
Informal business/Employee	13 (54.2%)
Housing situation	
Owner	5 (20.8%)
Renter	5 (20.8%)
Live with family/friends	11 (45.9%)
Squatter	3 (12.5%)
Average household family income, PhP	
0–10,000.00	16 (66.7%)
10,001.00–25,000.00	7 (29.1%)
25,001.00–40,000.00	1 (4.2%)

^a^ Distributions of variables are reported as n (%) unless specified otherwise.

**Table 2:** Effect of the type of delay and socio-demographic characteristics on the severity of dengue infection of patients admitted at NCH (n = 24).

Characteristics ^a^	Total (n = 24)	Severe Dengue Infection (n = 12)	Dengue with Warning Signs (n = 12)	Crude PR (95% CI) ^b^
Age of patient, years				
13–18 years old	8	2 (16.7%)	6 (50.0%)	1.00
0–12	16	10 (83.3%)	6 (50.0%)	2.50 (0.69–9.04)
Sex of patient				
Female	15	6 (50.0%)	9 (75.0%)	1.00
Male	9	6 (50.0%)	3 (25.0%)	1.67 (0.76–3.67)
Non-Quezon city residence				
No	15	5 (41.7%)	10 (83.3%)	1.00
Yes	9	7 (58.3%)	2 (16.7%)	2.33 (1.03–5.26) **
Relationship status of parent				
In a relationship	12	7 (58.3%)	5 (41.7%)	1.00
Single	12	5 (41.7%)	7 (58.3%)	0.71 (0.31–1.66)
Education of the parent				
At least a college student	9	2 (16.7%)	7 (58.3%)	1.00
None to high school graduate	15	10 (83.3%)	5 (41.7%)	3.00 (0.82–11.02) *
Religion				
Catholic	12	5 (41.7%)	7 (58.3%)	1.00
Non-Catholic	12	7 (58.3%)	5 (41.7%)	1.40 (0.60–3.24)
Occupation of the parent				
Informal business/Employee	13	6 (50.0%)	7 (58.3%)	1.00
None/Housewife	11	6 (50.0%)	5 (41.7%)	1.18 (0.52–2.67)
Housing situation				
Owner	5	2 (16.7%)	3 (25.0%)	1.00
Renter	5	3 (25.0%)	2 (16.7%)	1.50 (0.40–5.60)
Live with family/friends	11	5 (41.7%)	6 (50.0%)	1.14 (0.32–4.09)
Squatter	3	2 (16.7%)	1 (8.3%)	1.67 (0.42–6.54)
Average household family income, PhP				
0–10,000.00	16	10 (83.3%)	6 (50.0%)	1.00
<10,001.00	8	2 (16.7%)	6 (50.0%)	0.40 (0.11–1.45)
Decision time delay in proceeding to the health facility				
<1 h	15	8 (66.7%)	7 (58.3%)	1.00
≥1 h	9	4 (33.3%)	5 (41.7%)	0.83 (0.34–2.03)
Service delivery delay at the previous health facility				
Immediately/within 30 min	16	6 (50.0%)	10 (83.3%)	1.00
More than 30 min	2	1 (8.3%)	1 (8.3%)	1.33 (0.28–6.32)
More than 60 min	2	1 (8.3%)	1 (8.3%)	1.33 (0.28–6.32)
No service given	4	4 (33.3%)	0 (0.0%)	2.67 (1.40–5.09) **
Travel time delay to the current health facility				
Less than 1 h	7	4 (33.3%)	3 (25.0%)	1.00
1 h to less than 2 h	7	5 (41.7%)	2 (16.7%)	1.25 (0.56–2.81)
2 h or more	10	3 (25.0%)	7 (58.3%)	0.52 (0.16–1.69)
General travel time to the current medical facility				
Less than 30 min	11	6 (50.0%)	5 (41.7%)	1.00
30 min to less than 60 min	7	1 (8.3%)	6 (50.0%)	0.26 (0.04–1.81)
60 min to less than 120 min	4	4 (33.3%)	0 (0.0%)	1.83 (1.06–3.18) **
120 min or more	2	1 (8.3%)	1 (8.3%)	0.92 (0.20–4.19)

^a^ Distributions of variables are reported as n (%); ^b^ Crude Prevalence Ratio with 95% Confidence Interval; * *p*-value < 0.10 ** *p*-value < 0.05.

## Data Availability

The data are not publicly available due to privacy or ethical restrictions.
